# Glycaemic responses to liquid food supplements among three Asian ethnic groups

**DOI:** 10.1007/s00394-015-1072-1

**Published:** 2015-10-14

**Authors:** Siew Ling Tey, Ardy Van Helvoort, Christiani Jeyakumar Henry

**Affiliations:** 1 Clinical Nutrition Research Centre, Singapore Institute for Clinical Sciences, A*STAR, 14 Medical Drive, #07-02, Singapore, 117599 Singapore; 2 Nutricia Research, Uppsalalaan 12, 3584 CT Utrecht, Netherlands; 3 NUTRIM, School of Nutrition and Translational Research in Metabolism, Faculty of Health, Medicine, and Life Sciences, Maastricht University, Universiteitssingel 40, 6229 ER Maastricht, Netherlands; 4 Department of Biochemistry, Yong Loo Lin School of Medicine, National University of Singapore, 8 Medical Drive, Singapore, 117596 Singapore

**Keywords:** Glycaemic index, Glycaemic response, Liquid, Chinese, Indian, Malay

## Abstract

**Purpose:**

A limited number of studies have compared the glycaemic index (GI) and glycaemic responses (GR) to solid foods between Caucasians and Asians. These studies have demonstrated that Asians have greater GI and GR values for solid foods than Caucasians. However, no study has compared the GI and GR to liquids among various Asian ethnic groups.

**Methods:**

A total of forty-eight males and females (16 Chinese, 16 Indians, and 16 Malay) took part in this randomised, crossover study. Glycaemic response to the reference food (glucose beverage) was measured on three occasions, and GR to three liquids were measured on one occasion each. Liquids with different macronutrient ratio’s and carbohydrate types were chosen to be able to evaluate the response to products with different GIs. Blood glucose concentrations were measured in duplicate at baseline (−5 and 0 min) and once at 15, 30, 45, 60, 90, and 120 min after the commencement of beverage consumption.

**Results:**

There were statistically significant differences in GI and GR between the three liquids (*P* < 0.01 in all cases). However, there were no statistically significant differences in GI and GR for the liquids between the ethnic groups (Chinese vs. Indian vs. Malay).

**Conclusion:**

The GR for three different types of liquid nutritional supplements did not differ between the three main ethnic groups in Asia. It appears that the GI of liquid food derived from one Asian ethnicity can be applicable to other Asian populations.

## Introduction

Asia is currently experiencing a near exponential growth in diabetes and impaired glucose tolerance incidence rates. It is estimated that by 2025, more than 60 % of the world’s population of diabetics will reside in Asia [1]. These statistics highlight the urgent need to further understand the Asian genotype and develop means of controlling this growing pandemic.

Previous studies have shown that the glycaemic response (GR) to a standard food can differ between ethnic groups. Henry et al. [2] compared the GR to three types of biscuits and two types of cereal in Caucasians and South Asian Indians and showed that the latter group exhibited greater GR to these foods. In addition, Kataoka et al. [3] reported that Chinese demonstrated significantly greater GR to several rice varieties compared with Europeans. Whilst this is possibly due to differences in insulin sensitivity and beta-cell function [4, 5], these studies show the importance of considering ethnic differences, especially in those living in different regions of the world. There is evidence to show that South Asian Indians are more susceptible to impaired glucose tolerance possibly due to their body fat patterning favouring abdominal adiposity [6]. The rapidly increasing diabetes rates in Asians highlight the importance of individually considering this group and devising methods of reducing their diabetes risk.

Oral nutritional supplements are widely consumed in Asia and other parts of the world. They are drunk both to supplement normal food intake and as total meal replacers in situations where normal food intake is not possible or insufficient [7]. Oral nutritional supplements are a unique food group in that they are liquid-based and often have a complete macro- and micronutrient profile achieved by the incorporation of unique ingredients. There are many brands of nutrition supplements on the market with varying compositions. Some contain normal to high amounts of sugars for healthy individuals and those with compromised (reduced) nutritional intake, whilst others are low in sugars or contain slow-release carbohydrates suitable for diabetics or people at risk of hyperglycaemia. Earlier studies that report the metabolic response to standard oral nutritional supplements and disease-specific formulas mainly come from studies in Caucasians [8, 9]. It is important to understand whether these metabolic effects are applicable to Asians. This is an area that has not been investigated before. No studies have been conducted to compare the GR to oral nutritional supplements between different Asian ethnicities. Since glucose tolerance has been shown to differ between ethnic groups, it is possible that they also response differently to the nutrition formulas. Therefore the objective of this study was to compare the GR to three liquid nutritional supplements with different composition between Chinese, Indians, and Malay. These three ethnic groups represent over 60 % of the world’s population.

## Experimental methods

### Subjects

A total of 48 participants (16 Chinese, 16 Indians, and 16 Malay) with an equal number of males and females from each ethnic group were recruited from the general public in Singapore. The inclusion criteria were healthy males and females aged between 21 and 40 years, body mass index (BMI) between 18.5 and 25.0 kg/m^2^, fasting blood glucose <6.0 mmol/L, non-smoking, no metabolic, gastrointestinal or chronic diseases, no allergies or intolerances to the study liquids, no surgical events requiring hospitalization within the preceding 3 months, not taking prescribed medications known to affect blood glucose or body fat distribution, and not participating in sports at the competitive and endurance levels.

### Study procedures

The present study was conducted using a randomised, crossover design. Basic anthropometric measurements and blood pressure were taken using standard protocols during the first visit. The methodology used to measure the glycaemic index (GI) was adopted from that described by Brouns et al. [10] and was in line with the glycaemic response testing protocol recommended by the FAO/WHO [11] and the International Standards Organisation [12]. All participants were asked to attend six test sessions on non-consecutive days: in randomised order three sessions for testing the reference food (i.e. 50 g anhydrous glucose beverage) and another three sessions for testing the oral nutritional supplements. All test beverages were given in portions containing 50 g of available carbohydrates: beverage 1: 370 mL ready-to-drink Isocal, Nestlé Nutrition, Tainan, Taiwan; beverage 2: 427 mL ready-to-drink Diasip, Nutricia Advanced Medical Nutrition, Zoetermeer, The Netherlands; and beverage 3: 534 mL Protinex Diabetes Care powder mixed in milk according to label, Protinex, Mumbai, India. One hundred mL serving of beverage 1 provides 105 kcal, 3.4 g protein, 13.5 g carbohydrate (maltodextrin), and 4.4 g fat; beverage 2 provides 104 kcal, 4.9 g protein, 11.7 g carbohydrate (isomaltulose, starch, and lactose), 3.8 g fat and 2.0 g fibre; beverage 3 provides 98 kcal, 6.5 g protein, 9.4 g carbohydrate (lactose, maltodextrin, and starch), 3.4 g fat, and 1.7 g fibre (with 39 kcal, 3.5 g protein, 5.0 g carbohydrate, 0.1 g fat, and 1.7 g fibre from Protinex Diabetes Care powder). The blood glucose concentration in finger-prick capillary blood samples was measured using the HemoCue^®^ 201+ RT glucose analyser (HemoCue Ltd, Dronfield, UK). Participants arrived at the laboratory in the morning after a 10-h overnight fast. Following a 10-min rest, two blood samples were obtained 5 min apart for determining baseline blood glucose concentrations. They were then given either the reference food (glucose beverage) or the test beverage to consume. Further blood samples were obtained at 15, 30, 45, 60, 90, and 120 min for blood glucose measurements. Blood glucose measurements were performed by qualified researchers. Participants were asked to warm their hand to increase blood flow before a finger prick. Fingers were not squeezed to extract blood from the fingertips, but were gently massaged in order to minimize plasma dilution. The first two drops of blood were discarded, and the subsequent drop was used for testing.

This study was conducted according to the guidelines laid down in the Declaration of Helsinki, and all procedures involving human subjects were approved by the National Healthcare Group Domain Specific Review Board, Singapore. All participants gave written informed consent. This trial was registered at clinicaltrials.gov as NCT01889628.

### Statistical analysis

A sample size of 6–10 participants was recommended for glycaemic response studies in order to avoid type 2 errors [10, 11]. In order to provide 90 % power to detect an effect size of 0.35, a sample size of 16 was required for each ethnicity.

For each participant, the incremental area under the curve (iAUC) for blood glucose was calculated for both the reference food and test beverages. The total GR over 120 min was expressed as the iAUC ignoring the area under the baseline using the trapezoidal rule. In the case of the reference food, the mean of the three glucose tests was taken as the iAUC for the reference food. Using the iAUC values, the GI of the test beverages were calculated using the following formula [11]:

$${\text{GI}} = \frac{\text{iAUC for the test beverage}}{\text{Average iAUC for the reference food}} \times 100$$

One-way ANOVA was used to determine whether there were any statistical significant differences in baseline characteristics between the groups. The Scheffe test was used for the adjustment of multiple comparison tests. Linear mixed models with a random participant effect to account for the underlying correlation between the repeated measures were used to determine the effects of the interventions on the iAUC for blood glucose. The first model included a term ‘ethnicity’ for assessing the influence of the ethnic groups (Chinese, Indians, Malay) on iAUC for blood glucose, and the second model included the terms ‘ethnicity’ and ‘gender’. All statistical analyses were performed using Stata 11.2 (StataCorp, College Station, Tex, USA). Two-sided *P* < 0.05 was considered statistically significant in all cases.

## Results

### Participants’ characteristics

As shown in Table 1, participants were young with normal BMI and blood pressure. All baseline characteristics were well balanced between the ethnic groups, albeit a small but statistical significant difference was found in fasting blood glucose concentration (*P* < 0.001). The mean intra-individual CV for the reference glucose beverage for Chinese was 16.2 %, Indians was 19.0 %, and Malay was 18.7 %. These values were not statistically significantly different from each other (*P* = 0.538).

**Table 1:** Baseline characteristics of Chinese, Indians, and Malay

	Chinese (*n* = 16)	Indians (*n* = 16)	Malay (*n* = 16)	*P* value^*^
Age (year)	25.6 (3.0)	25.7 (2.1)	24.6 (3.6)	0.530
Height (cm)	169 (7.2)	169 (8.7)	163 (9.8)	0.086
Weight (kg)	60.4 (7.7)	60.1 (10.0)	58.1 (9.0)	0.730
Body mass index (kg/m^2^)	21.2 (1.9)	21.0 (2.0)	21.9 (2.1)	0.390
Systolic blood pressure (mm Hg)	108.6 (11.1)	101.1 (13.8)	103.4 (9.9)	0.190
Diastolic blood pressure (mm Hg)	67.2 (9.3)	65.4 (9.7)	62.7 (5.2)	0.315
Fasting blood glucose (mmol/L)	4.86 (0.5)^a^	4.15 (0.4)^b^	4.53 (0.4)^a, b^	<0.001

All values are means (SDs)

^*^One-way ANOVA models were used to determine whether there were statistically significant differences between the ethnic groups. The Scheffe test was used for the adjustment of multiple comparison tests. Means in a row without a common letter differ, *P* < 0.05

### Glycaemic response

**Fig. 1:**
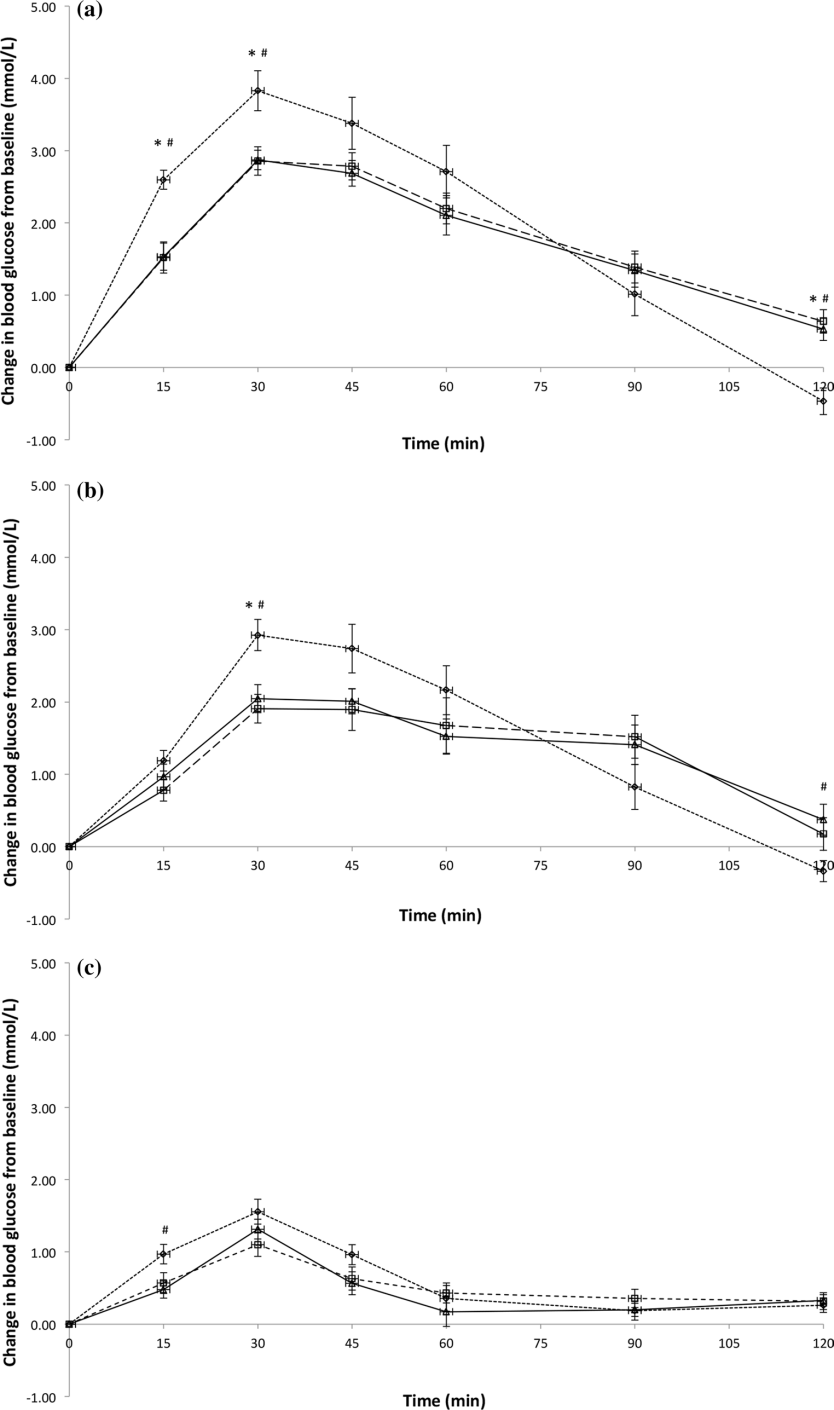

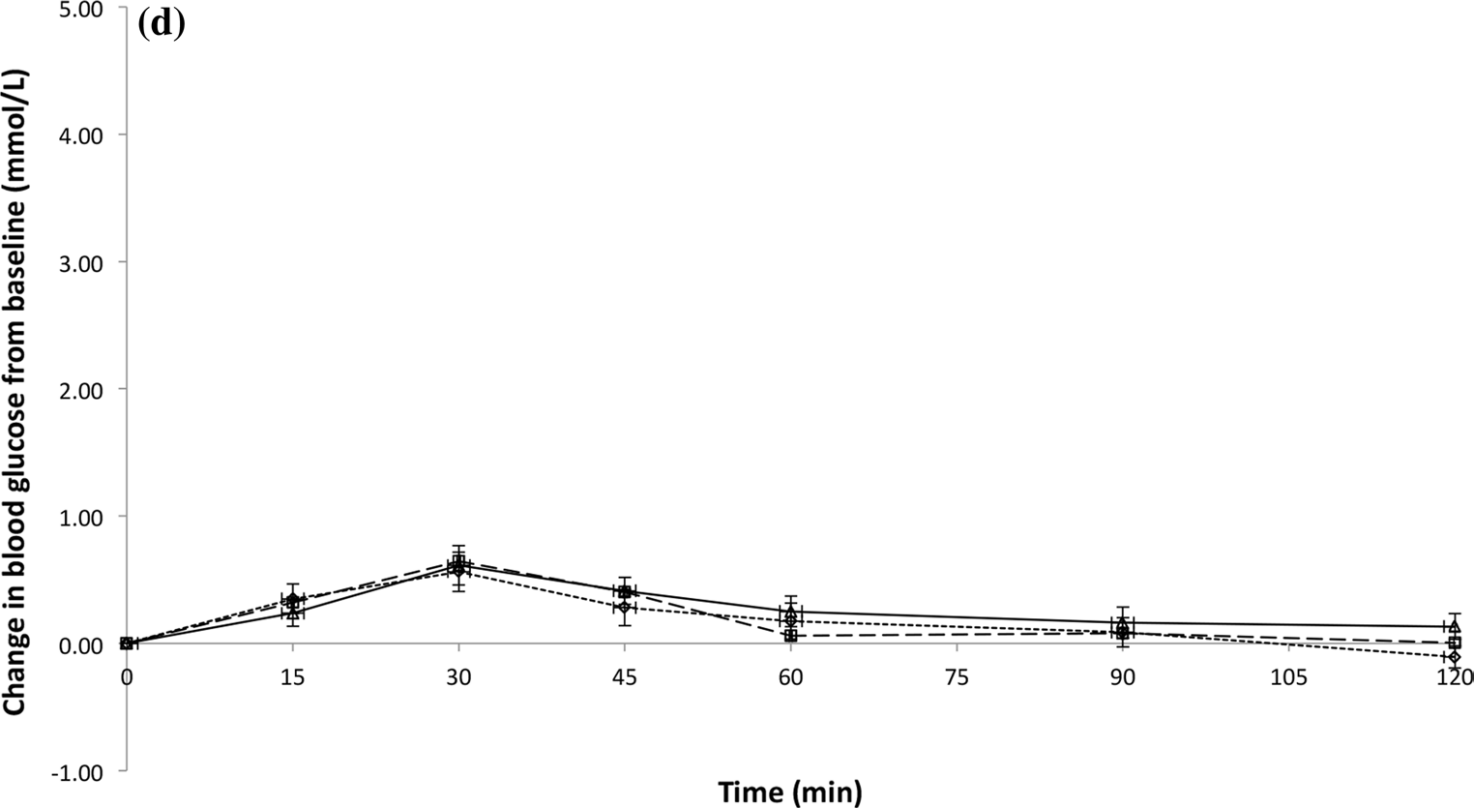
Temporal curves of the blood glucose response for glucose beverage (**a**), test beverage 1 (**b**), test beverage 2 (**c**), and test beverage 3 (**d**) among three ethnic groups. *Open diamond with dotted line* Chinese, *open square with dashed line* Indians, and *open triangle with solid horizontal line* Malay. *Mean values were significantly different between Chinese and Indians at a specified time point (*P* < 0.05). ^#^Mean values were significantly different between Chinese and Malay at a specified time point (*P* < 0.05)

Figure 1 shows the temporal GR patterns for the glucose beverage and all test beverages among three ethnic groups. There were statistically significant differences in the total iAUC for the GR over 120 min between the glucose beverage and all the test beverages (all *P* ≤ 0.003). The mean ± standard error iAUC for glucose beverage was 218 ± 11.8 (Chinese = 243 ± 25, Indians = 209 ± 19, Malay = 203 ± 17), test beverage 1 was 166 ± 10.8 (Chinese = 180 ± 19, Indians = 158 ± 23, Malay = 161 ± 13), test beverage 2 was 66 ± 4.8 (Chinese = 77 ± 8.7, Indians = 65 ± 7.8, Malay = 56 ± 8.3), and test beverage 3 was 35 ± 4.2 (Chinese = 35 ± 6.9, Indians = 33 ± 6.9, Malay = 38 ± 8.5) (Table 2). However, no statistically significant differences in total iAUC were found between the ethnic groups (*P* = 0.345). Results remained unchanged after adjusting for gender (*P* = 0.232) or fasting blood glucose at baseline (*P* = 0.175; data not shown) (Table 2).

**Table 2:** Glycaemic response and glycaemic index of the test beverages

	Chinese (*n* = 16)	Indians (*n* = 16)	Malay (*n* = 16)
Mean incremental blood glucose area under the curve (mmol/L min)			
Glucose beverage	243 (25)	209 (19)	203 (17)
Test beverage 1	180 (19)	158 (23)	161 (13)
Test beverage 2	77 (8.7)	65 (7.8)	56 (8.3)
Test beverage 3	35 (6.9)	33 (6.9)	38 (8.5)
Glycaemic index			
Test beverage 1	79 (8.8)	82 (13.3)	85 (8.5)
Test beverage 2	34 (4.1)	33 (5.5)	28 (3.5)
Test beverage 3	15 (2.8)	16 (2.9)	20 (4.8)

All values are means (SEs)

### Glycaemic index

There were statistically significant differences in the GI between the three test beverages (all *P* ≤ 0.003); the mean ± standard error GI for test beverage 1 was 82 ± 5.9 (Chinese = 79 ± 8.8, Indians = 82 ± 13.3, Malay = 85 ± 8.5), test beverage 2 was 32 ± 2.5 (Chinese = 34 ± 4.1, Indians = 33 ± 5.5, Malay = 28 ± 3.5), and test beverage 3 was 17 ± 2.1 (Chinese = 15 ± 2.8, Indians = 16 ± 2.9, Malay = 20 ± 4.8) (Table 2). However, there were no significant differences in GI of the test beverages between the ethnic groups (*P* = 0.964). Results remained similar after adjusting for gender (*P* = 0.346) or baseline blood glucose (*P* = 0.173; data not shown).

## Discussion

This study is the first trial of its kind to compare the glycaemic responses to three beverages varying in composition between three major Asian ethnic groups, namely Chinese, Indians, and Malay. Our study shows that the GR to the test beverages were not influenced by the ethnicity, which is in line with our previous study comparing the glycaemic responses to solid foods (i.e. Jasmine rice and Basmati rice) among various Asian ethnic groups [13]. Further to this, we found that gender of the participants did not affect GI and GR to the test beverages. This finding is in agreement with recent publication in this area [10]. It appears that the nutrient composition of the test beverages (e.g. type and relative amount of protein and carbohydrate as well as dietary fibre and the type of starch [10]), rather than the ethnicity or gender of the participants, plays an important role in determining glycaemic responses.

In conclusion, the GR to the test beverages did not differ between the ethnic groups, and thus the GI of the beverages did not differ between the study groups. Future studies should examine whether this finding can be generalized to other foods and also whether the finding derived from healthy Southeast Asians can be extrapolated to other Asian populations.
